# The Dual Role of *RASSF4* in Tumorigenesis: Mechanisms and Epigenetic Targeting Strategies

**DOI:** 10.3390/biology14091289

**Published:** 2025-09-18

**Authors:** Rui Tian, Yixin Wu, Wenbin Yuan, Lingli Tian, Rui Zhang, Hao Lyu, Shuai Xiao, Dong Guo, Qi Zhang, Declan William Ali, Marek Michalak, Cefan Zhou, Jingfeng Tang, Xing-Zhen Chen

**Affiliations:** 1 National “111” Center for Cellular Regulation and Molecular Pharmaceutics, Hubei University of Technology, Wuhan 430068, China; 18272297460@163.com (R.T.); heywyx2020@163.com (Y.W.); yuanwenbin@hbut.edu.cn (W.Y.); tian03132022@163.com (L.T.); zhangrui1987@hbut.edu.cn (R.Z.); leohnyy@foxmail.com (H.L.); xiaoshuai825@hotmail.com (S.X.); jk1103@whu.edu.cn (D.G.); zhangqi@hbut.edu.cn (Q.Z.); cefan@hbut.edu.cn (C.Z.); 2 College of Biological and Food Engineering, Hubei MinZu University, Enshi 445000, China; 3 Department of Biological Sciences, University of Alberta, Edmonton, AB T6G 2R3, Canada; dali@ualberta.ca; 4 Department of Biochemistry, University of Alberta, Edmonton, AB T6G 2R3, Canada; marek.michalak@ualberta.ca; 5 Membrane Protein Disease Research Group, Department of Physiology, Faculty of Medicine and Dentistry, University of Alberta, Edmonton, AB T6G 2R3, Canada

**Keywords:** RASSF4, methylation, tumor suppressor, epigenetic therapy

## Abstract

RASSF4 is a key member of the Ras-associated domain family (RASSF) that exhibits dual functionality in tumorigenesis, playing critical yet context-dependent roles in various malignancies. Its expression is epigenetically regulated through promoter hypermethylation, histone modifications, and microRNAs including miR-155 and miR-196a-5p, which directly target its 3′ untranslated region. In most cancers, such as non-small cell lung cancer (NSCLC) and gastric adenocarcinoma (GAC), RASSF4 acts as a tumor suppressor by inhibiting the RAS/MAPK pathway while activating the Hippo signaling cascade, ultimately inducing cell cycle arrest and apoptosis. Conversely, in aRMS, RASSF4 is upregulated by the PAX3-FOXO1 fusion oncoprotein and promotes tumor growth through MST1 inhibition and subsequent YAP activation. This review systematically analyzes current evidence regarding RASSF4’s complex regulatory mechanisms and clinical significance. We propose targeted therapeutic strategies including epigenetic reactivation, gene intervention, and combination therapies. Furthermore, we identify RASSF4 as a promising diagnostic biomarker and therapeutic target based on integrated mechanistic and clinical evidence. Future research should focus on elucidating context-dependent regulatory switches, developing targeted delivery systems, and validating clinical utility through prospective trials.

## Simple Summary

RASSF4 is a gene that plays two different roles in cancer depending on the context. In most cancers, it acts as a protective factor by slowing down cancer growth and triggering cell death. However, in a rare type of muscle cancer, it is hijacked to promote tumor development. This review explains how RASSF4 is controlled by chemical changes that switch it on or off, and how its behavior changes across cancer types. We discuss poten-tial treatments aimed at restoring RASSF4’s protective function in cancers where it is silenced, or blocking its harmful effects where it is overactive. We also highlight its promise as a diagnostic tool and a target for therapy. Our findings may help guide the development of more precise cancer treatments in the future, benefiting patients through improved therapeutic strategies.

## 1. Introduction

**Figure 1:**
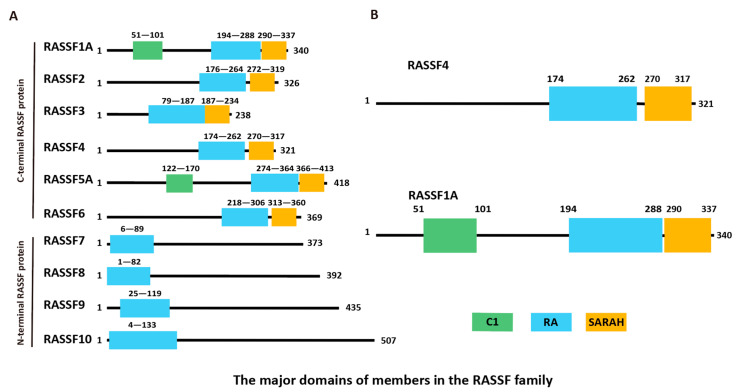
(**A**) Schematic diagram of the structure of the full-length gene sequences of RASSF family members. (**B**) Domains of RASSF4 and RASSF1A.

The mammalian RASSF proteins are designated RASSF1 to RASSF10 [1]. RASSF proteins serve as critical signaling scaffolds linking Ras GTPase activity to processes involving apoptosis, cell cycle arrest, and cell proliferation [2,3,4]. RASSF1 to RASSF6, referred to as C-terminal or classical RASSF proteins, possess the C-terminal RAS-associated (RA) domain and the Salvador–RASSF–Hippo (SARAH) domain (see Figure 1), with RASSF1 and 5A additionally containing the C1 domain [5]. The function of RASSF family proteins depends on their core domains such as RA, SARAH, and C1 (only unique to RASSF1A/5A). By mediating interactions with rat sarcoma viral oncogene homolog (RAS) GTPase, mammalian Ste20-like kinase (MST), and specific cell membrane phospholipids, these domains jointly determine the subcellular localization, transport of proteins, and their core functions in cell proliferation and apoptosis signal regulation [6,7]. RASSF7 to RASSF10 contain the N-terminal RA domain but lack the SARAH domain [8]. The RA domain is believed to interact with various RAS GTPases while the SARAH domain of RASSF1 to RASSF6 has been shown to promote the dimerization of other SARAH domain-containing proteins, including MST, a family of pro-apoptotic kinases [9,10,11].

RASSFs possess no reported enzymatic activity and likely function as adaptor or scaffold proteins in multiprotein complexes [4]. RASSFs are involved in various cellular activities, including apoptosis, cell cycle regulation, and microtubule stabilization [12,13]. Since the *RASSF* gene promoters are downregulated in various tumors through methylation modifications, RASSFs are considered tumor suppressors. In fact, in a variety of tumors, *RASSF1A*, *RASSF2*, *RASSF4*, *RASSF5*, and *RASSF6* were all found to be inactivated due to epigenetic modifications such as promoter methylation [14].

The human *RASSF4* gene is located on chromosome 10q11.21, contains 10 exons, and encodes a protein of 321 amino acids. RASSF4 is widely expressed in normal tissues [15,16]. RASSF4 is recognized as a tumor suppressor due to hypermethylation of its promoter and consequent transcriptional silencing in various cancers; however, its regulatory role is context-dependent and exhibits dual functions in different tumors. Exogenous overexpression of RASSF4 significantly inhibits cell growth and induces apoptosis, indicating that it has certain tumor suppressive activity [15,17,18]. However, in specific tumor types, high RASSF4 expression paradoxically promotes tumor growth by interacting with mammalian sterile 20-like kinase 1 (MST1) to inhibit the Hippo signaling pathway [19].

At present, therapeutic strategies targeting RASSF proteins mainly focus on using epigenetic drugs to reverse the hypermethylation of its promoter, thereby restoring its tumor suppressor function. In this field, the research on RASSF1A is relatively mature, especially the microcell-mediated chromosome transfer (MBR) adenovirus system strategy based on RASSF1A, modulator of apoptosis 1 (MOAP1), and Bcl-2-associated X protein (Bax) loading, showing significant potential in reversing drug resistance in solid tumors [20,21,22]. In contrast, RASSF4 is more challenging to study because of its environmental dependence on its expression and function—its cancer-suppressive function needs to be restored in most cancer types, while in some specific tumors, its pro-cancer activity needs to be suppressed [23]. Although this duality of function puts higher requirements on the individualization of treatment strategies, it also highlights the unique value of targeted RASSF4 in precision tumor treatment.

This review aims to summarize the molecular mechanisms of *RASSF4* as a key tumor suppressor gene and its multidimensional regulatory roles in tumorigenesis and progression, focusing on promoter methylation and the regulation of *RASSF4* expression by transcription factors. It subsequently explores core signaling pathways regulated by RASSF4 in the context of cell cycle regulation, apoptosis, and metastasis, notably the rat sarcoma/mitogen-activated protein kinase (RAS/MAPK) and Hippo–yes-associated protein (Hippo-YAP) pathways, while also evaluating the potential of *RASSF4* as a diagnostic and prognostic biomarker alongside its utility as a therapeutic target. It also summarizes novel approaches associated with gene therapy, epigenetic drug discovery, and combination therapy based on *RASSF4* restoration. By integrating the latest research advances, this review also proposes a framework for targeted tumor intervention involving *RASSF4*.

## 2. Cell Biological Processes Involving *RASSF4*

**Figure 2:**
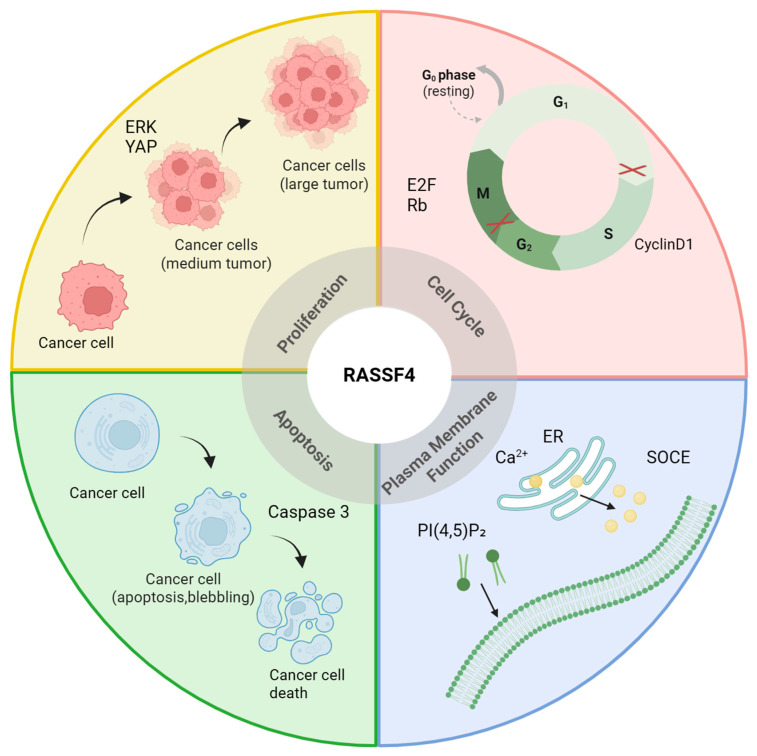
Core cellular biological processes regulated by *RASSF4*. *RASSF4* participates in the core regulatory mechanisms of multiple cellular biological processes, influencing tumor proliferation, apoptosis, cell cycle progression, and plasma membrane function. Created with BioRender.com.

*RASSF4* regulates cellular fate and homeostasis by coordinating processes such as apoptosis through caspase cascade activation, proliferation via inhibition of cyclin-D1, cell cycle arrest at the G1/S phase transition, fibrosis by suppressing hepatic stellate cell activation, and plasma membrane dynamics through ADP–ribosylation factor 6 (ARF6)–phosphatidylinositol 4,5-bisphosphate (PI(4,5)P2)-mediated signaling, thereby influencing the onset and progression of diseases, as shown in Figure 2.

### 2.1. Proliferation

Uncontrolled activation of proliferation is a core pathological mechanism driving tumorigenesis and fibrosis progression. It disrupts the balance of cell cycle regulation, promoting abnormal tissue proliferation and organ dysfunction, thereby accelerating the progression of malignant tumors [24,25]. *RASSF4* inhibits colorectal cancer (CRC) cell proliferation and colony formation by suppressing the Hippo–YAP signaling pathway and its downstream target B-cell lymphoma 2 (Bcl-2), while upregulating p21 expression, thereby blocking the cell cycle at the G1/S phase and inhibiting the malignant progression of CRC [2]. *RASSF4* inhibits osteosarcoma cell proliferation by regulating the Wnt–β-catenin signaling pathway. Its overexpression significantly reduces β-catenin, cyclin D1 (a key driver of the G1/S phase), and c-Myc protein levels, thereby arresting the cell cycle progression, ultimately inhibiting tumor cell proliferation and epithelial–mesenchymal transition (EMT) processes [26]. In metabolic fatty liver disease (MASLD), fatty liver inflammation (MASH), and hepatocellular carcinoma (HCC), transcriptomic analyses revealed significantly downregulated *RASSF4* expression. Overexpression of *RASSF4* can reduce the secretion of transforming growth factor β to inhibit the activation of hepatic stellate cells. Thus, the potential mechanism by which *RASSF4* regulates the progression of liver cancer is that *RASSF4* reduces transforming growth factor beta (TGF-β) secretion in hepatocytes, which inhibits the activation of hepatic stellate cells (HSCs) and then blocks abnormal proliferation of myofibroblasts and collagen deposition, thereby alleviating the progression of MASH to HCC [27]. In a study of NSCLC tissues. it inhibited proliferation by downregulating cyclin D1 to inhibit cell cycle progression [3]. *RASSF4* acts as a key tumor suppressor in multiple myeloma (MM), and its downregulation mediated by promoter methylation promotes tumor progression. Restoration of its expression inhibits malignant proliferation through a synergistic triple mechanism—inducing G2 phase cycle arrest to block mitotic progression, activating the MST1-JNK/c-Jun apoptotic pathway, and inhibiting the MEK/ERK and PI3K/mTOR/Akt survival signals, thereby significantly inhibiting MM cell proliferation [16]. In oral squamous cell carcinoma (OSCC), the high expression of miR-626 significantly downregulates the *RASSF4* expression, thereby activating the Wnt–β-catenin pathway to promote cancer cell proliferation and drive the malignant progression of OSCC through the EMT process [28]. Studies on gastric cancer (GC) indicate that in 75% of GC tissues, *RASSF4* is silenced and loses its inhibitory effect on proliferation. However, restoring the *RASSF4* function can induce G2/M phase cell cycle arrest (inhibiting DNA synthesis and mitosis), thereby inhibiting cancer cell proliferation and suppressing tumor growth [29]. However, *RASSF4* is significantly upregulated in alveolar rhabdomyosarcoma (aRMS), in which it inhibits the Hippo pathway (by inactivating MST1), leading to sustained activation of YAP1, which drives cell proliferation and escapes from senescence, representing a mechanism underlying the malignant progression in aRMS [19]. Studies also suggest that *RASSF4* inhibits mitogen-activated protein kinase (MAP kinase) signaling by suppressing the phosphorylation of extracellular signal-regulated kinase (ERK), providing another potential mechanism for *RASSF4*-mediated tumor suppression [30].

Tumor stem cells drive malignant tumor growth through their ability to continuously self-renew and indefinitely proliferate. They also resist radiotherapy and chemotherapy by leveraging their high DNA repair capacity and quiescent state, making them the root cause of tumor recurrence and resistance [31,32]. In oral cancer stem cells, overexpression of small proline-rich protein 1B (SPRR1B) significantly suppresses *RASSF4* protein levels, thereby inhibiting MAP kinase signaling-driven proliferation. Restoring the *RASSF4* function may emerge as a new therapeutic approach for interfering with cancer stem cells [30]. *RASSF4* acts as a key regulator of the cell cycle in mesenchymal stem cells (MSCs). Its downregulation relieves the inhibition of proliferation, driving the dedifferentiation of terminally differentiated cells and restoring their pluripotency and capacity for division. This provides a cellular resource for tissue regeneration [33].

In summary, *RASSF4* is a key regulator of cell proliferation. In most cases, as in most cancer types, *RASSF4* inhibits tumor proliferation through cycle arrest, but in some other cases (such as aRMS) it acts as a pro-cancer factor. Through its diverse functions, including coordinating the blockade of liver fibrosis and stem cell fate decisions, *RASSF4* serves as a central molecular switch maintaining tissue dynamic equilibrium.

### 2.2. Apoptosis

Apoptosis is an evolutionarily conserved form of programmed cell death that is critical for animal development and tissue homeostasis [34]. Apoptosis is closely associated with human diseases, such as the loss of apoptosis-inducing capacity being linked to cancer development [35]. A promising strategy in cancer therapy is to restore the apoptotic capacity of tumor cells. *RASSF4* regulates apoptosis through an environment-dependent dual mechanism; under stress or anti-cancer conditions (such as T-2 toxin exposure), its expression is activated by DNA demethylation and histone acetylation (H3K9ac), thereby promoting BCL2-associated X protein (BAX) activation and cytochrome C release and activating the caspase-9/3 cascade to induce apoptosis through the mitochondrial pathway [12,36]. Simultaneously, it synergistically enhances P38/JNK death receptor signaling and inhibits survival pathways such as PI3K-Akt-mTOR [16]. In GC, *RASSF4* overexpression activates the DNA damage response kinase Chk2, stabilizes p53 protein, and upregulates pro-apoptotic genes (such as *BAX* and *PUMA*), significantly promoting cancer cell apoptosis [29]. However, in some tumor microenvironments, *RASSF4* binds to MST1 via its SARAH domain to inhibit the core kinase cascade of the Hippo pathway (MST1–LATS1–YAP), blocking pro-apoptotic signals and promoting apoptosis resistance [37]. This functional duality is dynamically regulated by epigenetic modifications, inflammatory factors (such as the IL-6/DNMT1/Sp1 axis), and oncogenic mutations (such as the RAS–PI3K pathway), making *RASSF4* a bidirectional apoptosis regulator.

### 2.3. Functional Activities of the Plasma Membrane

As a dynamic signaling hub, the plasma membrane coordinates material transport, signal transduction, and cell adhesion to mediate communication between the intracellular and extracellular environments. Dysfunction of the plasma membrane is closely associated with pathological processes such as tumors and infectious diseases [38,39,40]. *RASSF4* plays a central regulatory role in this process; on one hand, it specifically binds to phosphatidylinositol-4-phosphate-5-kinase γ (PIP5KIγ) to drive the local production of PI(4,5)P_2_ [41], forming an “ARF6–PIP5KIγ–PI(4,5)P_2_” positive feedback axis to promote stromal interaction molecule 1 (STIM1) plasma membrane localization. On the other hand, it recruits endoplasmic reticulum–plasma membrane connection protein E-Syt2/3 to stabilize membrane contact sites [10], thereby reducing the spatial distance between STIM1 and calcium channel protein Orai1. These two pathways synergistically activate store-operated calcium influx (SOCE), significantly enhancing the calcium signaling efficiency. This mechanism not only provides a structural basis for *RASSF4*’s tumor suppressor function (such as maintaining contact-dependent growth inhibition) and Hippo pathway regulation, but also inhibits AKT phosphorylation, further weakening the host antiviral response during enterovirus 71 (EV71) infection, thereby enhancing viral replication [42], which highlights *RASSF4*’s regulatory potential as a cross-disease target.

## 3. Clinical Significance of *RASSF4*

### 3.1. As a Diagnostic and Prognostic Biomarker

*RASSF4* demonstrates significant potential as a diagnostic biomarker, primarily in terms of its epigenetic characteristics and tissue-specific expression. At the epigenetic level, its promoter region CpG island hypermethylation in tumors such as GC and head and neck squamous cell carcinoma (HNSCC) is closely associated with gene silencing, enabling non-invasive diagnosis via ctDNA liquid biopsy [43]. Additionally, the significant negative correlation between miR-626 and other miRNAs and *RASSF4* expression in oral squamous cell carcinoma provides auxiliary diagnostic value [28]; at the tissue expression level, low *RASSF4* protein expression in NSCLC is an independent prognostic marker in 41.57% of cases of lung adenocarcinoma and can be used for supplementary typing via immunohistochemistry [44]. Additionally, in NSCLC, reduced *RASSF4* levels are strongly linked to advanced tumor node metastasis (TNM) staging, lymph node metastasis, and poor prognosis. These findings suggest that *RASSF4* may function as an independent prognostic marker and a supportive indicator for diagnosis and treatment [3].

### 3.2. Role in Disease Progression

During the initiation stage of disease, *RASSF4* drives malignant transformation through epigenetic silencing: in precancerous lesions such as GC and HNSCC, the CpG island in its promoter region exhibits abnormal hypermethylation, with detection rates increasing from 28% in early stages to 67% in advanced stages, leading to transcriptional silencing [18,43]. A classic example is T-2 toxin exposure-induced hypermethylation of *RASSF4* in hepatocytes, which relieves inhibition of the PI3K-Akt/NF-κB pathway, triggering oxidative stress and apoptosis cascades [36]; simultaneously, transcriptional regulatory imbalance further blocks apoptosis checkpoints. In OSCC, overexpression of miR-626 targets and inhibits *RASSF4* translation, whereas in acute myeloid leukemia (AML), GATA-binding protein 2 (GATA2) significantly reduces *RASSF4* expression levels [29,45].

In tumor progression, dysregulation of core pathways drives malignant proliferation. *RASSF4* defects lead to inactivation of tumor suppression pathways. For example, in HCC, this defect inhibits MST1 activity while promoting YAP nuclear translocation. Similarly, in GC, impaired Chk2-p53 axis function weakens G2/M phase arrest. Conversely, *RASSF4* activation promotes oncogenic signaling, as observed in osteosarcoma, where it enhances β-catenin accumulation within the nucleus. In MM, *RASSF4* correlates with increased ERK phosphorylation, thereby accelerating cell cycle progression [16,26,27,29].

During the invasion and metastasis stage, microenvironmental remodeling promotes dissemination, with *RASSF4* regulating the extracellular matrix and immune microenvironment. Matrix metalloproteinase 2/9 (MMP2/9) secretion decreases by 65% in lung cancer, collagen deposition increases by 60% in stroke models, and the Treg proportion decreases by 40% and Th17 expansion increases in rheumatoid arthritis (RA) patients. In pancreatic cancer, it is positively correlated with programmed death-ligand 1(PD-L1) macrophage infiltration, forming a pro-metastatic microenvironment [19,44,46,47].

In the treatment resistance stage, drug resistance mechanisms form, with chemotherapy resistance arising from p53 pathway inhibition and enhanced drug efflux. In AML, p53 activity decreases by 65% and apoptosis decreases by 70%; in HCC, the IC50 of sorafenib increases. Targeted therapy escapes manifests as the response rate to YAP inhibitors dropping to 28%, while epigenetic drugs can restore *RASSF4* expression and increase cisplatin-induced apoptosis to 58%. Further studies on combined targeted therapy confirmed that restoring *RASSF4* expression using demethylating agents such as 5-Aza-2′-deoxycytidine enhances tumor cell sensitivity to cisplatin, suggesting that the *RASSF4* methylation status may serve as a potential biomarker for predicting responses to epigenetic therapy [28,45,48].

## 4. Roles of *RASSF4* in Tumorigenesis and Tumor Progression

**Figure 3:**
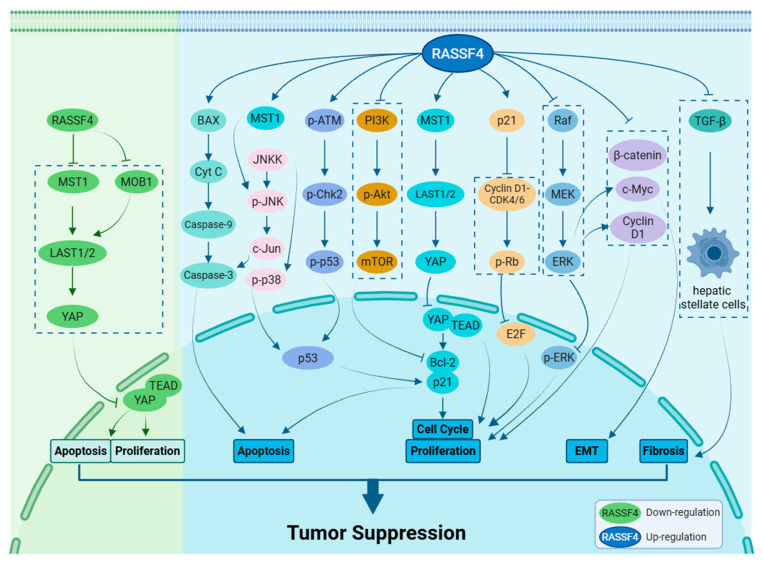
*RASSF4* molecular signaling. *RASSF4* participates in multiple signaling pathways, influencing tumor cell apoptosis, proliferation, cell cycle progression, EMT, and fibrosis. Ultimately, these effects impact the initiation and progression of cancer. Created with BioRender.com.

**Figure 4:**
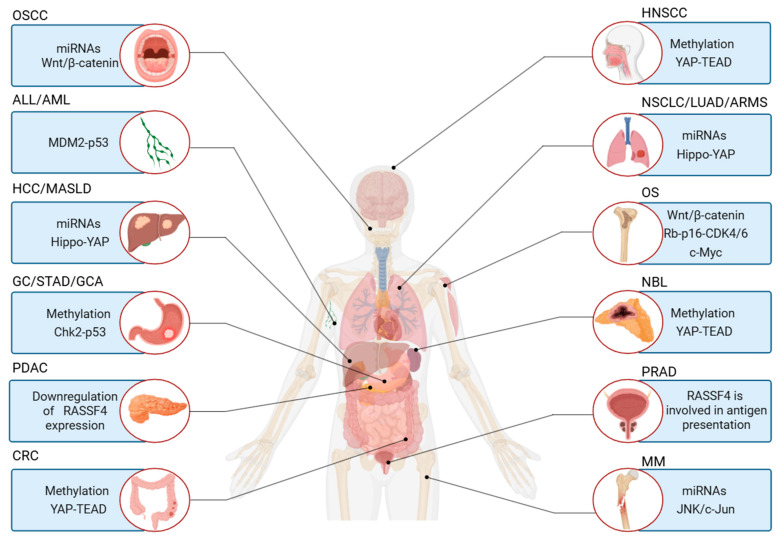
Mechanisms of *RASSF4* involvement in tumor regulation. *RASSF4* participates in the regulation of various tumors through distinct mechanisms, including methylation modification and non-coding RNA regulation, thereby influencing tumorigenesis and progression. Created with BioRender.com.

Tumorigenesis and tumor progression are complex biological processes involving multiple factors and stages. *RASSF4* plays an important role in these processes. It was reported that *RASSF4* participates in the development of various malignant tumors, including hepatic, gastric, and colorectal cancers, through regulating key signaling pathways such as RAS–MAPK and Hippo–YAP [27]. Additionally, its abnormal expression is significantly associated with clinical outcomes in diseases such as liver cancer and MM (see Figure 3 and Figure 4).

### 4.1. Mechanisms of How RASSF4 Inhibits Tumor Progression

*RASSF4* can function as either a tumor suppressor or an oncogenic activator, depending on the cellular context. In most cancer types, it primarily acts as a key tumor suppressor, inhibiting tumorigenesis and progression. Mechanistically, *RASSF4* suppresses tumor growth by modulating critical signaling pathways, including the Hippo, RAS/MAPK, and PI3K/AKT cascades, thereby influencing tumor cell fate [2,27,36]. In lung cancer, its reduced expression is significantly associated with poor prognosis, and restoring its expression can inhibit proliferation and expression of invasion-related key molecules (such as MMP2, MMP9, cyclin D1) and block clonogenic formation [3,44,49]. In GC, *RASSF4* induces cell cycle G2/M arrest and enhances chemotherapy-induced apoptosis by activating the Chk2–p53 axis, effectively inhibiting proliferation and enhancing chemotherapy sensitivity, indicating that *RASSF4* can be an important prognostic marker. In gastric cardia adenocarcinoma (GCA) and gastric adenocarcinoma (GAC), its downregulation is often associated with promoter methylation, and specific alternative splicing events in stomach adenocarcinoma (STAD) are associated with the prognosis and may influence the immune micro-environment [29,43,50]. In CRC, *RASSF4* inhibits malignant behavior by suppressing YAP/TEAD4-mediated Bcl-2 transcription and upregulating p21 [2]. *RASSF4* also inhibits the activation of hepatic stellate cells by reducing TGF-β secretion, thereby alleviating steatosis and fibrosis in MASLD. Additionally, it exerts anticancer effects by binding to MST1 to activate the Hippo pathway and inhibit the YAP nuclear translocation. Its deficiency promotes the progression of MASLD to HCC, while T-2 toxin-induced promoter hypermethylation relieves the inhibition of pathways such as PI3K–Akt, exacerbating hepatocyte damage [36].

Studies have confirmed that the *RASSF4* protein level is negatively correlated with the severity of MASLD and is an independent poor prognostic factor for HCC. *RASSF4* can serve as a biomarker for indicating MASLD disease progression and assessing HCC prognosis, and its associated pathways may provide new targets for targeted therapy in liver fibrosis and HCC [27,36]. In MM, low *RASSF4* expression predicts a poor prognosis, as it activates pro-apoptotic pathways (MST1, JNK/c-Jun, and p38) and inhibits pro-survival pathways (MEK/ERK and PI3K/mTOR/Akt), leading to cell cycle arrest and apoptosis and enhancing the efficacy of targeted drug therapy [16]. In osteosarcoma, its overexpression inhibits the Wnt/β-catenin pathway (downregulating β-catenin, cyclin D1, and c-Myc) to suppress proliferation, invasion, and EMT [26]. In OSCC, *RASSF4* as a tumor suppressor is targeted and inhibited by miR-626 and regulated by methylation. Restoring its expression can inhibit malignant phenotypes via blocking the Wnt/β-catenin pathway, and its expression level is an important prognostic indicator [28]. Downregulation of its expression has also been observed in neuroblastoma (NBL), and DNA methylation inhibitors can restore its expression [47].

It is worth noting that the function of *RASSF4* is environment-dependent. In some cellular models such as H1299 lung cancer cells, it may inhibit Hippo pathway activity through interaction with MST1, suggesting a potential tumor activator role. Additionally, *RASSF4* profoundly influences the tumor microenvironment and immune regulation. Specifically, deficiency or splicing abnormalities in *RASSF4* expression, as frequently observed in STAD, can reshape the immune landscape by altering immune cell infiltration and modulating key immune signaling pathways. In HNSCC, methylation of its promoter is associated with recurrence risk and has biomarker potential [18]. In conjunctival melanoma (CoM), deletion of the chromosome 10 region where it is located is a marker for metastasis risk [51]. In prostate cancer (PRAD), it has been identified as a potential tumor antigen associated with poor prognosis and specific immune cells, suggesting its potential as a target for mRNA vaccines [52]. A single-cell analysis of pancreatic ductal adenocarcinoma (PDAC) also identified it potentially as a key factor for the disease development [47]. In summary, *RASSF4* plays indispensable roles in the development and progression of various tumors by regulating core signaling pathways (such as RAS/MAPK, Hippo–YAP, Wnt/β-catenin, p53, and PI3K-Akt), the cell cycle, apoptosis, metabolism, and the immune microenvironment. Its widespread and potent tumor-suppressing effects indicate that it may be an important prognostic marker and a highly promising therapeutic target. However, the environmental dependency of its function, potential controversies of the studies, and its effects on the immune microenvironment indicate its involvement in complex tumor regulation, which should be considered in the development of targeted tumor treatment strategies.

### 4.2. Mechanisms by Which RASSF4 Promotes Tumor Progression

Although *RASSF4* exhibits tumor-suppressing effects in most tumors, it also demonstrates clear tumor-promoting functions in some tumor types such as aRMS. a highly aggressive childhood muscle-derived cancer in which the oncogenic fusion protein paired box 3–forkhead box O1 (PAX3-FOXO1) stimulates the *RASSF4* protein expression [53]. The core mechanism by which this upregulated *RASSF4* exerts its pro-tumorigenic effect lies in its inhibition of the Hippo signaling pathway; *RASSF4* binds to and inhibits MST1, a core tumor suppressor kinase in the Hippo pathway, thereby relieving MST1’s normal inhibitory effect on its downstream effector molecule YAP. The abnormal activation of YAP subsequently drives cell cycle progression, promotes cell proliferation, and helps tumor cells evade senescence checkpoints, which was validated in PAX3-FOXO1-positive aRMS cells and tumors [53]. Thus, this key oncogenic signaling axis in the development of aRMS can be depicted as ‘PAX3–FOXO1 → activation of *RASSF4* → inhibition of MST1 → activation of YAP’. Notably, *RASSF4* promotes myogenic differentiation (by activating the MST1–Hippo pathway), but in the carcinogenic context of aRMS, due to PAX3-FOXO1-driven activation, instead inhibits the MST1–Hippo pathway, thereby promoting tumorigenesis. Regarding CRC, a study found that among 118 patients with CRC, 73 (61.8%) showed significantly upregulated *RASSF4* expression through immunohistochemical detection [54]. This highlights the environmental dependency of *RASSF4*’s scaffolding function, with its oncogenic or anti-oncogenic effects depending on the specific cellular context and upstream driver signals [19,55].

### 4.3. RASSF4 Regulation of the Cell Microenvironment and Immunity

As a critical factor in cellular survival and function, the cell microenvironment provides a central pathological basis for disease onset and progression through disrupted homeostasis. In tumor biology, the tumor microenvironment (TME) modulates key biological processes such as immune evasion, angiogenesis, and extracellular matrix remodeling. This creates pathological conditions that are conducive to the malignant proliferation, invasion, and metastasis of tumor cells [56,57,58]. *RASSF4* as a key member of the RASSF family not only plays a central regulatory role in fundamental life processes such as cell proliferation, apoptosis, and signal transduction but also possesses significant potential for microenvironment regulation [29]. *RASSF4* deeply participates in the pathological process of malignant tumor progression by regulating intercellular communication and metabolism in the tumor microenvironment at multiple levels, making it a key molecular target related to tumorigenesis and development.

In terms of immune regulation, studies have found that *RASSF4* is a direct target of miR-99b-5p [46]. In RA, overexpression of miR-99b-5p inhibits the *RASSF4* expression, leading to a reduced T cell apoptosis rate and an increased proliferative index, and promotes the secretion of pro-inflammatory factors such as IL-2, IL-6, TNF-α, and IFN-γ, ultimately exacerbating joint inflammatory responses. This finding not only confirms the critical role of *RASSF4* in maintaining immune homeostasis but also provides a new targeted intervention strategy for RA treatment.

In terms of angiogenesis, studies have confirmed that *RASSF4* inhibits tissue repair through a triple mechanism [19]: (1) promoting the formation of a fibrotic microenvironment; (2) maintaining a pro-inflammatory state (sustained elevation of inflammatory factor levels); (3) antagonizing pro-angiogenic signals such as vascular endothelial growth factor (VEGF). This inhibitory effect on tissue repair is particularly pronounced in elderly individuals, with the vascular regeneration efficiency in patients over 65 years old reduced by approximately 70% compared to younger groups, providing a new molecular perspective on age-related tissue repair impairments. With respect to treatment resistance, AML studies have revealed a central role of the GATA2–*RASSF4*–MDM2–p53 signaling axis [45]. Experimental data showed that GATA2 overexpression reduces the *RASSF4* mRNA level by 80%, leading to a threefold increase in MDM2 activity and a 65% inhibition of p53 expression. A clinical sample analysis revealed that patients with low *RASSF4* expression levels have a complete remission rate of only 28.6%, significantly lower than the high-expression group (71.4%). Combining doxorubicin with the MDM2 inhibitor Nutlin-3 can reverse this drug-resistant phenotype, increasing the treatment response rate to 82.3%. These findings not only elucidated the central role of *RASSF4* in regulating the tumor microenvironment but also provided important evidence for developing targeted therapeutic strategies.

### 4.4. The Potential Influence of Tumor Microenvironment Characteristics on RASSF4 Functions

Korz et al. reported that *RASSF4* was specifically upregulated in cognitively unimpaired aged rats (OMG group), which also showed enrichment in pathways such as estrogen signaling and complement activation [59]. The complement system (C4a, C4b) is a key inflammatory mediator. This association implies that *RASSF4* may be involved in adaptive or protective responses to age-related inflammation, possibly modulating cell survival or synaptic function in a neuroinflammatory context. Additionally, Buga et al. found that *RASSF4* was persistently upregulated in aged stroke rats and was associated with fibrotic scar formation—a process driven by TGF-β and inflammatory cytokines such as IL-6 and IL-18. Although not directly tested, the persistence of *RASSF4* expression in pro-fibrotic and inflammatory environments suggests it may be regulated by inflammatory cytokines (e.g., via TGF-β or STAT signaling) and could contribute to pathological remodeling in response to chronic inflammation [60].

### 4.5. The Relationship Between Genetic Changes in Tumor Cells and the Functioning of This Protein

Although RAS mutations are frequently observed in MM and drive oncogenic signaling through pathways such as MEK/ERK and PI3K/AKT, Eva et.al indicate that the tumor-suppressive function of *RASSF4* remains intact irrespective of RAS mutational status. Enforced expression of *RASSF4* induced cell-cycle arrest and apoptosis in both RAS-mutated and wild-type cell lines. Furthermore, no significant correlation was observed between *RASSF4*-mediated pro-apoptotic effects and the presence of NRAS or KRAS mutations in primary MM samples. These results suggest that *RASSF4* acts as a RAS effector capable of activating pro-death pathways such as MST1/JNK/c-Jun signaling independent of RAS activation status. Thus, epigenetic loss of *RASSF4* may enable tumor cells to evade RAS-induced growth suppression, thereby promoting malignant progression. Therapeutic strategies aimed at restoring *RASSF4* expression could, therefore, be beneficial even in RAS-driven MM [16].

## 5. Regulatory Mechanisms of the *RASSF4* Expression

**Figure 5:**
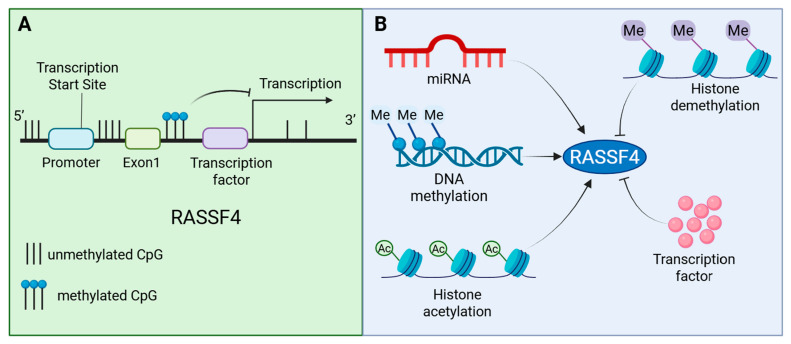
Regulation of *RASSF4* expression: (**A**) schematic diagram of methylation sites within the *RASSF4* gene and their corresponding transcriptional regulatory regions; (**B**) epigenetic regulation enhances the transcriptional activity of *RASSF4* through mechanisms such as non-coding RNA, DNA methylation, and histone acetylation modifications, all of which occur without altering gene sequences. Concurrently, histone deacetylation and transcription factors can suppress *RASSF4* transcription. Created with BioRender.com.

*RASSF4* plays a critical role in tumorigenesis and progression, with its expression and activity being precisely regulated through multi-tiered mechanisms, including epigenetic, transcriptional, and translational processes. Among these, epigenetic regulation processes, such as DNA methylation, histone modifications, and non-coding RNAs, modulate *RASSF4* expression without altering its DNA sequence [15,28,36] (see Figure 5). Additionally, the subcellular localization of *RASSF4* significantly influences its functional outcomes. These regulatory mechanisms work in concert to collectively shape the pivotal role of *RASSF4* in tumor development.

### 5.1. Epigenetic Regulation

#### Epigenetic Regulation of *RASSF4* in Cancer

The expression of *RASSF4* is regulated through multiple mechanisms, among which DNA methylation represents a pivotal epigenetic pathway. This regulatory mechanism dynamically influences tumor progression and significantly modulates the tumor-suppressive function of *RASSF4* [43,49]. In various malignant tumors, the CpG islands enriched in the 5′ promoter region of the *RASSF4* gene often undergo abnormal methylation modifications, a process primarily mediated by DNA methyltransferases 1 (DNMT1) and DNMT3A/B [61,62], leading to transcriptional silencing of the gene. This methylation state is directly associated with the two opposite effects of *RASSF4*: (1) when the promoter region is hypomethylated, *RASSF4* is normally expressed and exerts its tumor-suppressing effect by activating the RAS-dependent apoptosis pathway; (2) in contrast, hypermethylation leads to a reduced *RASSF4* level, which in turn triggers abnormal activation of the Hippo–YAP pathway and excessive activation of the RAS/MAPK signaling pathway within cells, driving the malignant progression of tumors.

Notably, the *RASSF4* methylation level exhibits significant spatiotemporal heterogeneity. Studies on GC indicate that the methylation content of specific CpG sites within the CpG-enriched exon 1 region is closely correlated with the mRNA expression level. An analysis of the public database (DepMap) revealed a significant negative correlation between *RASSF4* mRNA expression levels and DNMT3A protein expression. This finding suggests that DNMT3A may catalyze abnormal hypermethylation of CpG sites in GC, leading to epigenetic silencing of *RASSF4* transcription. This may be an important mechanism underlying the frequent inactivation of *RASSF4* in GC [29]. Dynamic monitoring of CRC shows that the methylation rate is 28% in the early stages of the disease and rises to 67% in the late metastatic stage, suggesting that the methylation process is positively correlated with tumor invasiveness. Multiple studies have further confirmed that *RASSF4* hypermethylation frequently occurs in recurrent HNSCC [18], NBL, GCA, and other tumors, with methylation levels negatively correlated with gene expression. Additionally, 12.5% of nasopharyngeal carcinoma (NPC) cell lines exhibit *RASSF4* deletion, with highly methylated promoter regions, and demethylating agents can restore its mRNA expression [15,43,48]. Environmental factors such as T-2 toxin exposure can also induce high methylation of the *RASSF4* promoter, leading to its low expression [49]. However, in some tumors (such as pheochromocytoma, bladder cancer, and thyroid tumors), *RASSF4* inactivation is not significantly associated with DNA methylation [54,63,64], indicating that the silencing effect of DNA methylation on *RASSF4* is cell environment-dependent. This dependency may be related to differences in specific transcription factors regulating *RASSF4* (such as ZF5, Pax-5, and AHR) across different cancers and their interactions with DNMT, which promote site-specific methylation [49,65].

In summary, DNA methylation modifications represent a core epigenetic mechanism regulating the expression status and functional activity of *RASSF4*. Its dynamic changes directly determine the switching between tumor suppression and promotion functions of *RASSF4* in tumors. As shown in Table 1, DNA methylation modifications participate in tumorigenesis and progression by regulating key gene expression, holding critical significance for understanding tumor development mechanisms and exploring targeted intervention strategies.

In addition to DNA methylation, histone modifications and non-coding RNAs are also key epigenetic mechanisms regulating the *RASSF4* expression [49]. Studies have shown that while no significant enrichment of repressive histone modifications (H3K9me3 and H3K27me3) was observed in XG-7 (IL-6-dependent cell line) and MM1S cells, treatment with the histone deacetylase inhibitor (HDACi) quisinostat significantly upregulates *RASSF4* protein expression in human MM cell lines (AMO-1 and JJN3) [16], suggesting that a low acetylation state in the *RASSF4* promoter region may inhibit its transcription. Additionally, in the RA model, overexpression of miR-99b-5p was shown to directly target and inhibit *RASSF4* expression, thereby promoting abnormal proliferation and activation of synovial cells [46]. Recent studies in OSCC have also found that abnormally high expression of miR-626 can directly target and inhibit the translation of *RASSF4* mRNA, and the expression of the two shows a significant negative correlation, indicating its key role in the occurrence and development of OSCC [28]. In studies of NSCLC, it has been established that miRNA-155/miRNA-196a-5p directly binds to the 3′-UTR of *RASSF4*, negatively regulating its mRNA level [44]. In summary, the histone acetylation status and the expression levels of some miRNAs together regulate the *RASSF4* protein expression. These epigenetic mechanisms play a crucial role in the occurrence and development of various diseases such as MM, RA, and OSCC, and their dysregulation directly affects cellular behavior and drives disease progression.

### 5.2. Transcriptional Regulation and Subcellular Localization Regulation

The expression regulation of *RASSF4* not only depends on epigenetic modifications but also on transcriptional, translational, and post-translational regulation. At the transcriptional regulation level, *RASSF4* expression is known to be inhibited by the stem cell transcription factor GATA2, and high expression of GATA2 down-regulates the *RASSF4* protein level [45]. Earlier studies first revealed that *RASSF4* serves as a direct target of the bHLH transcription factor Atoh1, confirming that Atoh1 not only activates neuron-specific gene expression but also negatively regulates the *RASSF4* protein expression [66]. Subsequently, Michifuri et al. found that the proline-rich small protein SPRR1B negatively regulates the *RASSF4* expression through a mechanism yet to be determined [30]. Further, during the development of aRMS, overexpressed PAX3-FOXO1 significantly up-regulates both the *RASSF4* mRNA and protein levels, thereby promoting tumorigenesis and progression [19]. These findings collectively reveal a complex transcriptional regulatory network for *RASSF4* under different physiological and pathological conditions.

## 6. Therapeutic Potential and Future Directions for Targeting *RASSF4*

**Figure 6:**
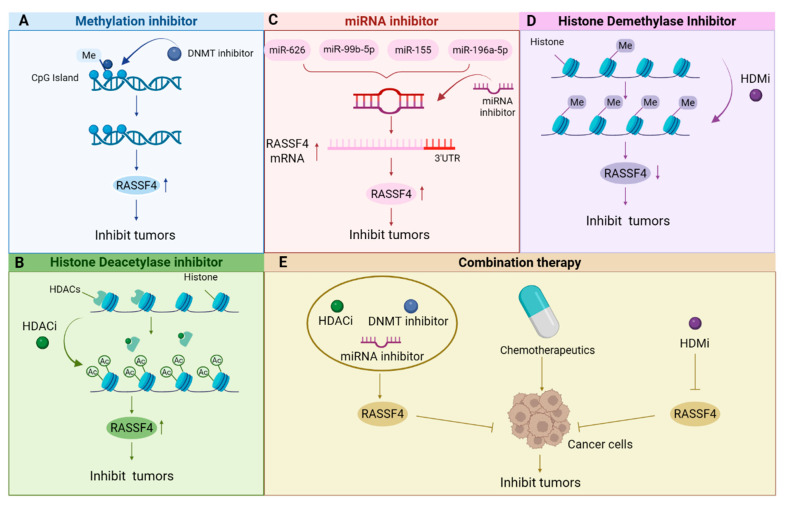
Therapeutic strategies targeting the dual regulatory functions of *RASSF4*: (**A**) reversing the epigenetic silencing of *RASSF4* by targeting DNA methyltransferases (DNMTs); (**B**) transcription activation of *RASSF4* by regulating histone acetyltransferases; (**C**) modulating *RASSF4*-related signaling networks using miRNA-based therapeutic approaches; (**D**) inhibiting histone demethylases to downregulate *RASSF4* expression; (**E**) *RASSF4*-guided precision combination therapy, combining gene therapy with chemotherapy drugs. Created by BioRender.com.

Building upon the established regulatory mechanisms underlying *RASSF4* dysregulation, this section examines potential therapeutic approaches aimed at restoring its tumor-suppressive functions. Given the documented roles of promoter hypermethylation and miRNA-mediated downregulation in *RASSF4* silencing, promising strategies may involve: (1) epigenetic reactivation; (2) miRNA-mediated regulation of *RASSF4* and therapeutic prospects; (3) direct genetic intervention via viral or non-viral vectors to deliver and restore *RASSF4* gene expression; (4) combining pathway-specific drugs to enhance *RASSF4*-mediated tumor suppression (as shown in Figure 6). Table 2 summarizes potential *RASSF4*-targeted therapeutic agents. The following discussion evaluates the preclinical evidence supporting these strategies and highlights critical challenges in their translational development.

### 6.1. Epigenetic Reactivation Strategies

Epigenetic modifications play a key role in tumorigenesis and progression [69,70,71]. Among these, DNA methylation controls gene expression by affecting DNA transcription. This process is primarily mediated by DNA methyltransferases (DNMTs), which catalyze the transfer of a methyl group (-CH_3_) from the donor S-adenosylmethionine to the 5′ position of the cytosine residue in DNA, particularly within CpG islands [72,73,74,75]. DNA methylation is often associated with gene silencing. In many malignant tumors, abnormal DNA methylation promotes tumor growth and survival [76,77,78,79]. In various tumors, the silencing of *RASSF4* expression is frequently associated with hypermethylation of its promoter region and abnormal histone modifications [29,45]. However, studies have shown that epigenetic regulatory drugs could reverse this process. Demethylation drugs inhibit the activity of DNA methyltransferases, reduce DNA methylation, restore normal gene expression patterns, and thereby induce tumor cell differentiation or apoptosis [75,80]. Evidence indicates that DNA demethylating drugs (e.g., 5-aza-2′-deoxycytidine and decitabine) inhibit DNMT enzyme activity, leading to a marked upregulation of *RASSF4* expression. It is suggested that this mechanism could have a therapeutic benefit, as improved treatment responses have been observed in NBL and liver injury models [48].

In addition to DNA methylation, histone modifications are also important epigenetic mechanisms that influence gene expression states and drug sensitivity [81]. Research confirms that HDACi quisinostat and paibastat can effectively restore *RASSF4* expression in MM cells by regulating epigenetic modifications. In vitro experiments demonstrated that treating human myeloma cell lines (HMCLs), such as XG-7, AMO-1, and JJN3, with HDACi significantly upregulated *RASSF4* mRNA and protein levels, with particularly pronounced effects in cells exhibiting low baseline *RASSF4* expression. This finding was validated in vivo using the 5T33MM mouse model, where quisartinib monotherapy or combination with decitabine significantly increased *RASSF4* expression in tumor cells. Further functional studies demonstrated that combining HDACi with the MEK inhibitor trametinib significantly synergistically enhanced anti-myeloma effects. Combined treatment induced stronger apoptosis, demonstrating high synergy. Both panitistam and trametinib treatment induced *RASSF4* upregulation, highlighting the crucial role of histone modifications in regulating *RASSF4* expression. This suggests that targeting HDAC and MEK in combination offers a promising new therapeutic approach for future treatments [16,82,83].

In aRMS, the PAX3-FOXO1 fusion protein recruits histone demethylase (KDM3B), which reduces H3K9 methylation levels and promotes the expression of the oncogenic target gene PAX3-FOXO1 [53]. Evidence suggests that PAX3-FOXO1 binds to the *RASSF4* promoter, leading to enhanced transcriptional activity. The subsequent upregulation of *RASSF4* may thereby drive tumor growth [19]. Consequently, KDM3B inhibitors suppress tumor growth by inhibiting demethylase activity. This leads to the accumulation of H3K9me2 methylation marks, which silences oncogenes and exerts an antitumor effect. This passive promotion of methylation’ strategy is an important paradigm in targeted epigenetic therapy, and demonstrates particular breakthrough potential in PAX3-FOXO1 fusion-positive rhabdomyosarcoma.

With the rapid development of precision medicine, the use of demethylating agents has become an important strategy in cancer therapy [84,85,86]. These drugs reverse abnormal DNA methylation in tumor cells, reprogram gene expression profiles, and induce tumor cell differentiation and apoptosis [87,88,89]. Recent studies have characterized the spatiotemporal dynamics of DNA methylation following demethylating agent treatment at single-cell resolution. These analyses provide clear evidence to elucidate the mechanisms of epigenetic regulation [90].

Currently, commonly used clinical demethylating drugs such as decitabine and azacitidine can significantly reduce genomic methylation levels, but their clinical application still faces three major challenges: drug toxicity issues, including gastrointestinal reactions, bone marrow suppression, and increased risk of opportunistic infections; therapeutic heterogeneity manifested as significant differences in response rates across different tumor types and even subtypes of the same tumor; drug resistance issues, which manifest as long-term use leading to the reconstruction of methylation patterns and the activation of drug metabolism enzymes [91,92,93]. In response, ongoing research is investigating potential strategies such as novel nanocarrier-based delivery systems to improve targeting, combination therapies with immune checkpoint inhibitors or other targeted agents, and biomarker-guided treatment utilizing methylation profiles to anticipate sensitivity. These approaches may better facilitate the broader and more precise application of demethylating agents in oncology.

### 6.2. miRNA-Mediated Regulation of RASSF4 and Therapeutic Prospects

In addition to DNA methylation, histone acetylation and microRNA (miRNA) also participate in the regulation of *RASSF4* expression. Further, miRNAs represent a class of endogenous non-coding RNAs with lengths of 21–23 nucleotides that regulate gene expression by binding to mRNA [94,95,96]. They participate in various biological processes such as cell proliferation, differentiation, and apoptosis, and play a critical regulatory role in the development and progression of numerous diseases, including cancer, cardiovascular diseases, and neurodegenerative disorders [97,98,99]. In terms of mechanism, miRNAs bind to the 3′ untranslated region (3′UTR) of target mRNAs through incomplete complementary binding, thereby inhibiting the expression of target genes. Studies have shown that in NSCLC, OSCC, and RA, specific miRNAs can negatively regulate the expression levels of *RASSF4*, thereby influencing disease progression and prognosis. Specifically, these miRNAs directly target the 3′UTR of the *RASSF4* mRNA, substantially inhibiting *RASSF4* protein expression, and this inhibitory effect is notably positively correlated with the malignancy of the disease. Notably, in different pathological types, such NSCLC and OSCC, the degree of reduced *RASSF4* expression is closely associated with clinical pathological features such as the tumor stage and metastatic potential. In patients with RA, low *RASSF4* expression is also significantly correlated with disease activity and joint damage severity. These findings suggest that the miRNA have confirmed. *RASSF4* regulatory axis may serve as a potential therapeutic target and prognostic marker for the aforementioned diseases [28,44,100]. Due to the multi-target nature of miRNAs [101], they possess unique advantages in disease treatment but also increase the risk of off-target effects.

Small-molecule drugs have long been the preferred treatment for cancer [102]. Additionally, miRNA can simultaneously intervene in complex disease pathways (such as tumors and metabolic diseases) by regulating multiple target genes, and is regarded as an “innovative drug” following small molecules and antibody drugs. Related research is currently a hot topic in the field of biomedicine, covering multiple areas such as tumors, fibrosis, metabolic diseases, and cardiovascular diseases. However, no miRNA drugs have been approved for marketing worldwide at present [98,103]. Based on regulatory mechanisms, miRNA drugs can be categorized into two main strategies: inhibiting overexpressed miRNAs and supplementing or activating underexpressed miRNAs. The first category blocks the binding of miRNA to target mRNA, thereby downregulating abnormally expressed miRNA. This primarily includes miRNA antagonists (antagomirs) [104,105,106,107], miRNA sponges [108,109,110,111], and miRNA masks [111,112]. The second category involves supplementing underexpressed miRNAs, including miRNA mimics. The mimics of miRNA are synthetic molecules that mimic endogenous miRNAs. They compensate for the loss of miRNA expression in diseased cells to restore normal function [113,114].

Developing a safe and efficient delivery system for miRNA therapy is a key challenge in its clinical application. Current delivery strategies are primarily divided into two major categories: viral vectors and non-viral vectors. In the viral vector field, AAV has become one of the most commonly used viral vectors in gene therapy due to its low integration risk and sustained expression of exogenous genes. Currently, three investigational drugs utilize viral vectors, namely the AAV9, AAVrh, and AAV5 delivery systems [115]. Non-viral vector delivery systems primarily include lipid nanoparticles, iron oxide nanoparticles, and enzyme-directed vector technology microscopic (EDV^TM^) nanocells [116,117]. Similar to other small nucleic acid drugs, how to achieve tissue-specific delivery while reducing miRNA’s rapid degradation and drug side effects remains a pressing issue.

Therapeutic strategies targeting *RASSF4* have formed a multi-tiered integrated system, involving epigenetic reactivation, miRNA intervention, and gene therapy delivery systems. Early-stage lesions can be treated with epigenetic drugs combined with miRNA inhibitors for preventive therapy, while advanced tumors are suitable for *RASSF4* gene replacement combined with targeted drugs. Three key breakthroughs are needed in the future: developing precise epigenetic editing tools, optimizing targeted delivery systems, and establishing a precise classification system based on methylation or miRNA. These advancements will drive the application of *RASSF4*-targeted therapy in precision medicine for tumors.

### 6.3. Direct Gene Delivery for RASSF4 Restoration

Epigenetic regulation and miRNA intervention can restore *RASSF4*’s function, but these indirect regulatory methods still have obvious shortcomings in terms of expression accuracy and sustainability. In contrast, gene intervention strategies that directly target *RASSF4* achieve precise gene delivery and stable expression through viral and non-viral vector systems, providing more direct and effective treatment options for patients with advanced tumors and metabolic diseases.

Studies have shown that abnormal downregulation of *RASSF4* expression is closely associated with the onset and progression of various diseases [27,50]. According to the latest research progress, gene intervention requires the use of two complementary treatment strategies. First, for disease types with low *RASSF4* expression, including most solid tumors and advanced patients with metabolic diseases, viral vectors such as AAV or lentivirus, or non-viral vectors such as GalNAc-modified lipid nanoparticles, can be used to directly deliver the *RASSF4* gene to restore its tumor-suppressing function [118,119]. It is worth noting that the heterogeneous and dynamic characteristics of the tumor microenvironment, including hypoxia, acidic pH, and immune suppression, significantly affect the drug delivery efficiency. Therefore, researchers are developing novel delivery systems, such as pH-sensitive and enzyme-sensitive responsive carriers, which can markedly enhance targeting efforts and effectively overcome treatment resistance [120,121,122].

On the other hand, for specific pathological states driven by *RASSF4* overexpression, such as certain cancer subtypes, RNA interference technology or CRISPR-Cas9 gene editing systems are required to specifically inhibit *RASSF4* expression [123]. Extensive research has shown that siRNA can silence genes essential for tumor initiation and progression. Moreover, it offers advantages over conventional chemotherapy drugs, including a shorter development cycle, high efficacy at low doses, fewer side effects, and lower resistance [124,125]. The CRISPR gene knockout system facilitates the precise knockout of specific genes by inducing frameshift mutations via non-homologous end-joining repair. This technology has been widely applied in studying the genetic functions of various tumor and hereditary diseases [126,127]. Based on extensive research, CRISPR/Cas9 therapy has entered the clinical trial phase, with its feasibility and safety already validated in patients with NSCLC [128,129]. The successful implementation of these treatment strategies relies on the targeted optimization of delivery systems to ensure therapeutic efficacy while minimizing potential adverse effects.

In summary, gene intervention strategies that directly target *RASSF4* by precisely regulating its expression levels offer new insights into the treatment of various diseases. Future research should focus on optimizing the targeting and safety of delivery systems to advance these therapeutic strategies from laboratory research to clinical application.

### 6.4. Combination Therapy and Pathway Synergy Strategies

While monotherapy targeting *RASSF4* has demonstrated some efficacy, given *RASSF4*’s regulatory role across multiple signaling pathways, combination therapy and pathway-synergistic drug strategies are emerging as new research directions.

The synergistic use of gene therapy and chemotherapy drugs has characteristics such as simultaneous blockade of multiple pathways, complementary therapeutic targets, and increased drug sensitivity [130,131,132,133,134].Combination therapy and pathway-synergistic strategies targeting *RASSF4* have been explored in preclinical studies, and *RASSF4* overexpression significantly enhances tumor cell sensitivity to traditional chemotherapy drugs. In NSCLC models, *RASSF4* gene therapy combined with cisplatin produces a synergistic effect by activating the p53 pathway to induce tumor cell apoptosis. Similarly, in liver cancer treatment, *RASSF4* overexpression can reverse tumor resistance to 5-fluorouracil. In GC, combination with 5-FU results in a significant reduction in tumor volume [2,27,29,44]. In p53-deficient models, the combination of *RASSF4* activators with MDM2 antagonists (such as Nutlin-3) significantly improves the complete remission rate in AML [45]. The synergistic effect of *RASSF4* overexpression and multiple targeted drugs can better inhibit further tumor progression. As a key regulator of the RAS/MAPK and Hippo pathways, *RASSF4* exhibits synergistic effects with various targeted drugs. Combination with MEK inhibitors enhances the inhibitory effect on KRAS-mutant tumors, while co-administration with YAP inhibitors synergistically inhibits tumor metastasis; in tumors with abnormal activation of the PI3K/AKT pathway, *RASSF4* overexpression enhances the efficacy of PI3K inhibitors [27,28,49].

Therapeutic strategies targeting *RASSF4* have formed a multi-level integrated system, involving epigenetic reactivation, miRNA intervention, and gene therapy delivery systems. Early-stage lesions can be treated with epigenetic drugs combined with miRNA inhibitors for preventive therapy, while advanced tumors are suitable for *RASSF4* gene replacement combined with targeted drugs. Three key challenges that must be addressed in the future include developing precise epigenetic editing tools, optimizing targeted delivery systems, and establishing a precise classification system based on methylation or miRNA. These advancements will drive the application of *RASSF4*-targeted therapy in precision oncology.

## 7. Discussion and Conclusions

### 7.1. Discussion

The most striking feature of *RASSF4* is its highly context-dependent functionality. In specific settings such as aRMS, it exerts oncogenic effects by inhibiting the Hippo pathway, challenging the traditional theory of single-function tumor suppressor genes. Its double-edged nature indicates that its ultimate role in cancer progression depends on complex interactions between genetic, epigenetic, and microenvironmental factors.

It should be particularly noted that current therapeutic strategies targeting *RASSF4* are primarily based on theoretical predictions derived from its molecular mechanisms and research findings, lacking systematic preclinical trial data to support them. These potential approaches encompass epigenetic reactivation, gene interventions, and combination therapies, whose feasibility and efficacy require validation through rigorous preclinical studies.

Future research studies face several critical challenges and directions. Firstly, the precise molecular switches driving *RASSF4* dual functionality must be elucidated, including its interactome, post-translational modifications, and upstream signaling drivers. Secondly, clinical translation of *RASSF4* requires overcoming challenges such as drug delivery, tumor heterogeneity, and resistance. Developing novel targeted delivery systems including nanocarriers and exosomes alongside rational combination therapies such as with immune checkpoint inhibitors or YAP pathway inhibitors represents a crucial direction. Thirdly, it is imperative to explore the deeper mechanisms of *RASSF4* within the tumor microenvironment, particularly its regulation of immune cell function, metabolic reprogramming, and intercellular communication. This will open new avenues for combined immunotherapy. Furthermore, a deeper understanding of how the tumor microenvironment influences *RASSF4*’s function will be essential for developing effective targeted therapeutic strategies in the future. Finally, advancing biomarker-based precision patient stratification and conducting well designed clinical trials are key to validating its clinical safety and efficacy, extending its application to rare cancers and even benign diseases.

In summary, *RASSF4* represents a significant regulatory target whose substantial diagnostic and therapeutic value remains to be fully explored. While challenging, it undoubtedly constitutes a highly promising target within precision oncology, offering critical opportunities for innovative therapeutic strategies.

### 7.2. Conclusions

*RASSF4* plays a complex and multifaceted role in tumor biology. This review systematically summarizes the current research, indicating that in most cancers, *RASSF4* functions as a tumor suppressor by inhibiting the RAS/MAPK pathway and activating the Hippo pathway, thereby suppressing cell proliferation and promoting apoptosis. Its expression is finely regulated by DNA methylation, histone modifications, and microRNAs such as miR-155 and miR-196a-5p, with promoter hypermethylation being a central mechanism for its silencing. The expression and methylation statuses of *RASSF4* demonstrate significant potential as diagnostic and prognostic biomarkers in various cancers, including gastric carcinoma, NSCLC, and MM. Therapeutic strategies targeting *RASSF4*, including epigenetic reactivation, gene therapy, and combination therapies, represent key future research directions.

## Figures and Tables

**Table 1:** Aberrant DNA methylation of *RASSF4* and related genes across different cancers.

Cancer Types	Cell Lines	Methylation Factors	Genomic Regions Involved	References
Head and neck squamous cell carcinoma	Hep-2, RPMI-2650, and UM-SCC-14C	*MST1*, *RARβ*, *MLH1*, *DAPK*, *p16*, *RASSF5*, *MGMT*	The CpG island promoter regions of *RASSF2* and *RASSF4*	[18]
Neuroblastoma	Kelly, NB69, SK-N-SH, SH SY-5Y, SK-N-AS, SK-N-BE (2), SK-N-DZ, SK-N-FI, and IMR-32	*RASSF5*, *RASSF6*, *RASSF7*, *RASSF2A*, *RASSF4*, *RASSF8*, and *RASSF10*	The CpG island promoter regions of *RASSF5A*, *RASSF5C*, *RASSF6*, *RASSF7*	[48]
Gastric cancer	SNU16, SNU216,	*RASSF4*	The exon 1 of *RASSF4*	[29]
	SNU484, SNU601,			
	SNU620, SNU638,			
	SNU719, MKN1,			
	MKN28, AGS,			
	MKN74, MKN45,			
	KATOIII			
Stomach adenocarcinoma	MKN-45, and AGS	—	The exon regions of *CD44*, *RASSF4*, *PPP2R5D*, and *LOH12CR1*; the terminator regions of *PPHLN1* and *CADPS*; the alternative promoter regions of *KIAA1147*, *CDKN3*, and *WEE1*	[50]
Gastric cardia adenocarcinoma	—	*RASSF*, *RASSF3*,	The exon 1 of *RASSF2*, *RASSF3*, *RASSF4*, and *RASSF6*	[43]
		*RASSF4*, and		
		*RASSF6*		
Colorectal cancer	LoVo, HCT-8, HCT116, and HCT15	*RASSF4*	The promoter regions of Bcl-2	[2]

**Table 2:** Prospective pharmaceuticals targeting *RASSF4* in cancer treatment.

Type	Potential Representative Drug	Target	Cancer	Potential Mechanism	Reference
Epigenetic regulatory drugs	Azacitidine	DNA CpG islands	OSCC HNSCC	Demethylating	[28] [18]
	Decitabine		GCA LUAD NSCLC		[43] [49] [67]
	EGCG				
	Vorinostat	Histone acetyltransferase	M M	Histone deacetylase inhibitors	[16]
	Panobinostat				
Gene-targeted therapeutic drugs	dCas9-TET1 fusion system		GC CRC	Gene activation /editing	[29] [2] [49]
	SunTag-TET1 multivalent system	*RASSF4*	LUAD		
	miRNA sponge vector	miRNA	NSCLC LUAD OSCC	miRNA antagonist	[44] [49] [28]
	LNA-antimiR-626				
	Cholesterol-modified antagomiR-155				
	Locked nucleic acid (LNA) or GalNAc–siRNA conjugate	*RASSF4* splicing variants	STAD	siRNA	[50]
Signal pathway synergistic inhibitor	Verteporfin	YAP	aRMS OSCC CRC HNSCC	Inhibition of YAP–TEAD binding	[19] [28] [2] [18]
	XMU-MP-1	MAT1/2	HCC MM aRMS	Activate MST1/2	[27] [49] [19]
	Trametinib	MEK	M M	RAS/MAPK	[49]
	Sotorasib	KRas		Inhibition of KRAS activity	[68]
